# mARC1 Is the Main Contributor to Metabolic Reduction of *N*-Hydroxyurea

**DOI:** 10.1021/acs.jmedchem.4c01148

**Published:** 2024-10-14

**Authors:** Cathrin Klopp, Xiaomei Zhang, Morgan K. Campbell, David Kvaskoff, Michel A. Struwe, Curtis R. Warren, Besnik Bajrami, Axel J. Scheidig, Amanda K. Jones, Bernd Clement

**Affiliations:** 1 Zoological Institute − Structural Biology, Kiel University 24118, Kiel, Germany; 2 Pharmaceutical Institute − Medicinal Chemistry, Kiel University 24118, Kiel, Germany; 3 Department of Cardiometabolic Disease Research, Boehringer Ingelheim Pharmaceuticals, Inc., Ridgefield, Connecticut 06877, United States; 4 Department of Drug Discovery Sciences, Discovery Science Technologies (DK, BB), Boehringer Ingelheim Pharma GmbH & Co. 88400, Biberach an der Riss, Germany

## Abstract

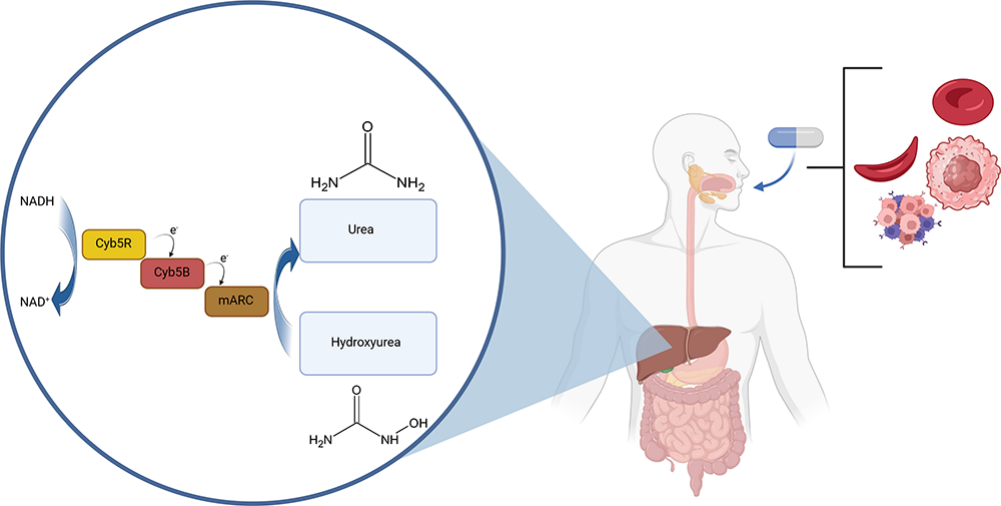

*N*-Hydroxyurea has been known since the 1960s as an antiproliferative drug and is used both in oncology and for treatment of hematological disorders such as sickle cell anemia where very high daily doses are administered. It is assumed that the cellular effect of *N*-hydroxyurea is caused by inhibition of ribonucleotide reductase, while alternative mechanisms, e.g., generation of nitric oxide, have also been proposed. Despite its many therapeutic applications, the metabolism of hydroxyurea is largely unexplored. The major elimination pathway of *N*-hydroxyurea is the reduction to urea. Since the mitochondrial amidoxime reducing component (mARC) is known for its *N*-reductive activity, we investigated the reduction of NHU by this enzyme system. This study presents *in vitro* and *in vivo* evidence that this reductive biotransformation is specifically mediated by the mARC1. Inactivation by mARC1 is a possible explanation for the high doses of NHU required for treatment.

## Introduction

*N*-Hydroxyurea (NHU), also known as hydroxycarbamide, is a well-known drug substance that has been approved for use in different types of cancer for more than half a century. The antitumor activities of NHU were first discovered in the 1960s when Stearns *et al.* found the substance to be effective against leukemia.^1^ The main mechanism of NHU antiproliferative activity is believed to be the inhibition of ribonucleotide reductase (RNR), catalyzing the last step in the synthesis of DNA building blocks and removal of the 2′-hydroxy group at the ribose moiety. This is presumably achieved by scavenging a catalytically important tyrosyl radical at the enzyme’s active site.^2,3^ RNR is essential for the synthesis of deoxyribonucleotides from ribonucleotides. Consequently, inhibition of RNR leads to the decreased synthesis of DNA.^4^ Alternative mechanisms for the inhibition of cell replication by NHU have been the subject of a recently reviewed article.^5^

The first patients with chronic myelogenous leukemia (CML) were already treated with NHU in the 1960s, so the proliferation-inhibiting effect has been exploited for a long time.^6^ NHU is also approved for use in nononcological diseases including sickle cell anemia (SCA). The first clinical trial showing increased fetal hemoglobin and total hemoglobin in patients with SCA upon administration of NHU was reported in 1984,^7^ and a number of clinical trials have confirmed the beneficial effects of NHU treatment in SCA since.^8^ Administration of NHU is indicated for patients with multiple episodes of severe acute pain or acute chest syndrome,^9^ as these complications of SCA are reduced significantly by NHU administration.^10^ Long-term studies further showed that in addition to a decrease in painful episodes and acute chest syndrome, NHU is able to reduce mortality in patients with SCA.^11,12^ For treatment of SCA, orphan drug status has been granted to NHU in both the United States and the European Union, where it is sold under the commercial names Hydrea and Xromi, respectively.^13^

The exact mechanism by which NHU exerts its positive influence on fetal hemoglobin production in patients with SCA is not completely understood. Possible mechanisms include nonspecific interruption of the cell cycle by inhibition of RNR and nitric oxide (NO) signaling.^9^

Potential mutagenic effects of NHU treatment have long been a clinical concern. However, neither preclinical nor clinical trials show any mutagenic effect of NHU at pharmacologically relevant doses.^14^

NHU is also used in other therapeutic areas like the hematological disease β-thalassaemia.^15^ Studies on rodent models furthermore suggest a potentially beneficial effect of NHU in Alzheimer’s disease (AD).^16−18^

**Figure 1:**
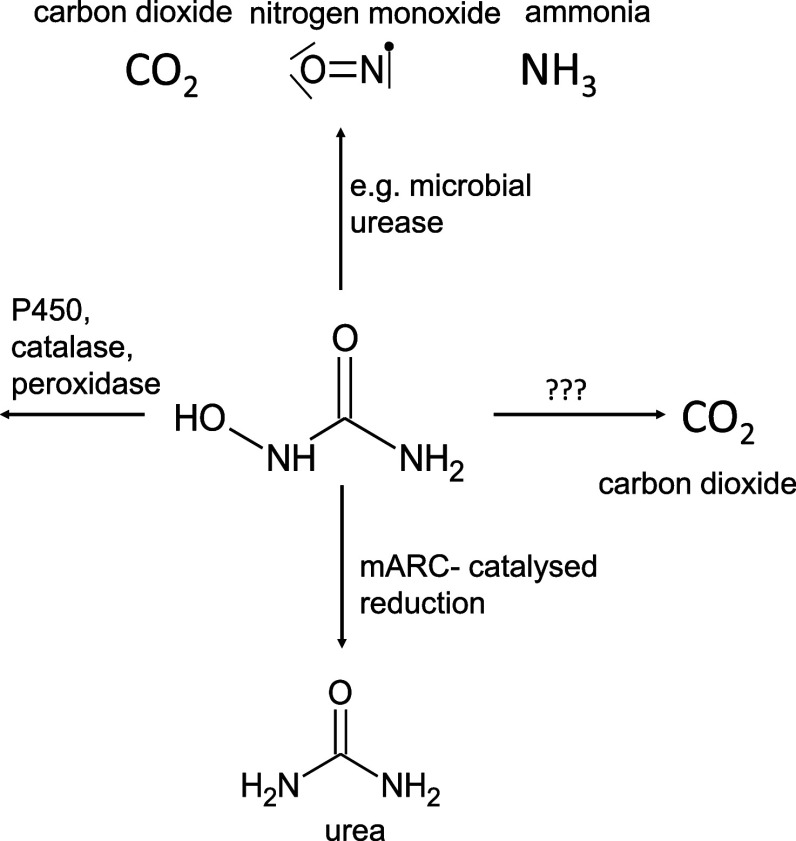
Elimination pathways of NHU-known enzymatic conversions are those by urease, peroxidase, catalase, and P450. In addition, a conversion to urea is known, which can be attributed to the mARC enzyme system.

Although different biotransformation pathways of NHU have been identified previously, the specific enzymes involved in these pathways have remained unknown. The enzymes known to be involved in the conversion are shown in Figure 1.

Understanding the inactivation and elimination pathways of therapeutic drugs is important, as it allows to adjust the administered dosage or recognize potentially unfavorable pharmacokinetic interactions with other drugs.^19^ Here, we investigate the main metabolic inactivation pathway for NHU, the two-electron reduction toward urea, and identify the enzyme responsible.

It is currently known that orally administered NHU is highly bioavailable with the distribution volume roughly equivalent to the total body water, suggesting that tissue and protein binding of NHU are negligible. However, the drug is eliminated quite rapidly with reported half-life times averaging at approximately 2–4 h.^20^ NHU doses for SCA treatment are often escalated to the highest tolerated dose, which was found to be superior to a fixed-dose scheme, with effectiveness usually ranging from 15 to 30 mg × kg^–1^ × day^–1^.^21^

Different elimination pathways were studied in 1965 using ^14^C-labeled NHU in rodent models. Less than 1% radioactivity was observed in the feces, while approximately 7% were expired as carbon dioxide (CO_2_). While CO_2_ can be formed from NHU by the microbial enzyme urease, expiration of ^14^C-labeled carbon dioxide was found to be unchanged in germ-free mice, indicating that the microbiome is not involved in this conversion.^22^ However, the vast majority of ^14^C-radioactivity is recovered in the animals’ urine, with a small portion corresponding to carbonate, while the remaining ^14^C was identified as either nonmetabolized NHU or urea in approximately equal quantities (each amounting to >30% of the administered dose).^22^ Colvin and Bono Jr. then reported that NHU was reduced to urea directly in murine liver mitochondria through an unknown enzyme.^23^

Since carbon dioxide was also found to arise from NHU in germ-free mice, this indicates that additional processes not involving (microbial) urease might play a role. Fishbein *et al.* describe the conversion of NHU by urease to carbon dioxide, ammonia, and hydroxylamine, which is further oxidized to nitric oxide.^24^ Peroxidases, catalases, and P450 monooxygenases may be involved in the metabolism.^25^

A likely candidate for reduction of NHU to urea is the mitochondrial amidoxime reducing component (mARC), of which all mammalian genomes encode two paralogues, mARC1 and mARC2 (HUGO gene identifiers *MTARC1* and *MTARC2*). mARC enzymes are part of a three-component enzyme system together with cytochrome b5B (CYB5B) and NADH-cytochrome b5 reductase 3 (NB5R3) that catalyzes two-electron reduction of various *N*-hydroxylated compounds.^26^

Human mARC enzymes were originally discovered in the early 2000s due to their central role in activation of *N*-hydroxylated prodrugs of amidine-containing drug substances, *i.e*., inhibitors of serine proteases involved in the coagulation.^27,28^ The first FDA-approved representative of novel oral anticoagulant (NOACs), ximelagatran, relies on mARC for release of the active form melagatran after intestinal absorption.^29^

The human mARC enzymes have been investigated extensively over the past years, and many *N*-oxygenated compounds have been found to be mARC substrates.^26,28^ In general, the two mARC paralogues have largely overlapping substrate specificities, with very few compounds displaying a strong specificity for either mARC1 or mARC2. An exception are *N*-oxides, which are reduced exclusively by mARC1.^30,31^

In recent years, mARC enzymes have again become of great interest due to the potential involvement in lipid metabolism and pathogenesis of liver diseases associated with hepatic accumulation of triglycerides, *i.e.*, metabolic dysfunction-associated steatotic liver disease (MASLD) and metabolic dysfunction-associated steatohepatitis (MASH).^32−34^ Although the depletion of mARC1 in the liver unambiguously alleviates lipid accumulation and MASLD-related symptoms,^35^ its exact role in lipid metabolism remains unclear, and multiple pharmaceutical companies have now registered patents for liver-specific siRNA-based knockdown of mARC1.^28^ The first clinical trial investigating the safety, tolerability, and pharmacokinetics of a siRNA-based drug targeting mARC1 in hepatocytes has already been initiated (NCT05599945).^36^

In this study, we show that NHU is specifically reduced to urea by mARC1, while the contribution of mARC2 toward NHU reduction is likely negligible. This is demonstrated by both *in vitro* enzyme activity assays and by *in vivo* studies using murine *Mtarc1* knockout and hepatocyte-specific knockdown models. So far, no individual NHU-metabolizing enzyme has been described. This is the first study to identify mARC1 as an enzyme responsible for the biotransformation of NHU based on *in vitro* and *in vivo* studies. The high specificity of this reaction for mARC1 rather than mARC2 suggests the possibility to use NHU reduction as an *in vivo* biomarker for mARC1 activity, which was the second goal of this study.

## Results

NHU was first recognized as a potential mARC substrate by Indorf *et al.*(37) We have conducted more detailed investigations into the kinetics of NHU reduction using recombinant human mARC enzymes and complemented these *in vitro* studies with *in vivo* studies using murine models.

**Figure 2:**
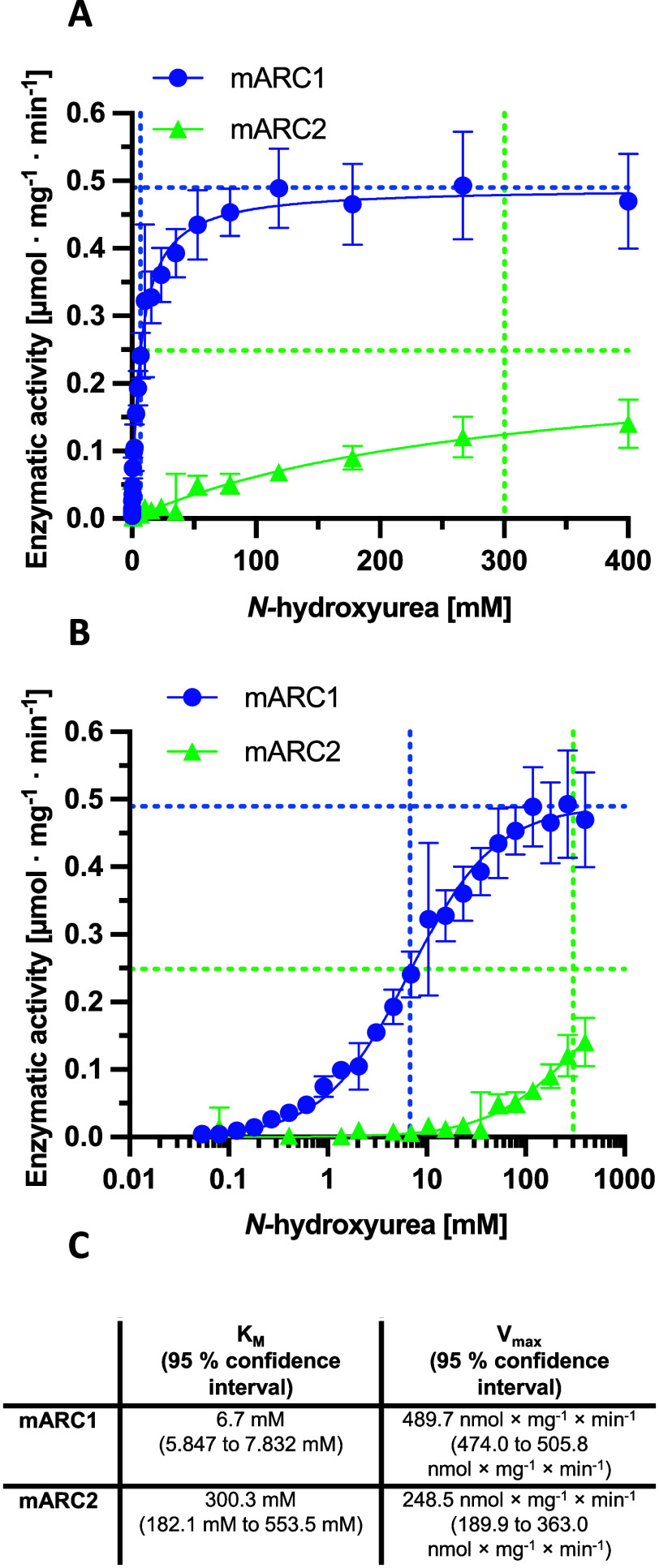
Michaelis–Menten kinetics for the reduction of *N*-hydroxyurea by human mARC1 and mARC2. (A) Linear plot. (B) Logarithmic (Boltzmann-like) plot. Dashed vertical lines represent *K*_M_ values; dashed horizontal lines represent *V*_max_ values. (C) *K*_M_ and *V*_max_ values. Assay conditions are described in the Materials and Methods section. Standard deviations are calculated from *n* = 6. As mARC2 activities were not yet saturated at the highest NHU concentrations, we examined that the *K*_M_ and *V*_max_ values are only rough estimates based on extrapolation.

Only mARC1 reduces NHU with kinetic parameters that we consider to be relevant in a biological context. Using our previously described *in vitro* activity assay with recombinant mARC1, mARC2, CYB5B, and NB5R3 proteins,^38^ we demonstrate that recombinant human mARC1 can, in concert with its electron transport partners CYB5B and NB5R3, reduce NHU with kinetics obeying the Michaelis–Menten equation. The *K*_M_ value of this reduction is approximately 7 mM, while the *V*_max_ was determined to be approximately 0.49 μmol × mg^–1^ × min^–1^. In contrast, recombinant mARC2 shows a much lower activity. From fitting to the Michaelis–Menten equation, the *K*_M_ value is estimated to be approximately 300 mM and *V*_max_ approximately 0.25 μmol × mg^–1^ × min^–1^. However, the fitted values for NHU reduction by mARC2 have high uncertainty, as the enzyme was not nearly saturated, even at the highest NHU concentration tested in our assays. This means that no reliable fit can be generated from the data, only a rough estimate. Regardless, at biologically relevant concentrations, mARC1 and not mARC2 would be the enzyme responsible for the reduction of this substrate. Michaelis–Menten curves are plotted in Figure 2.

As we consider the conversion rates of NHU by mARC2 too low to be of relevance at concentrations that could occur biologically, only mARC1 was investigated in *in vivo* studies.

### mARC1 *InVivo* Expression Systems

**Figure 3:**
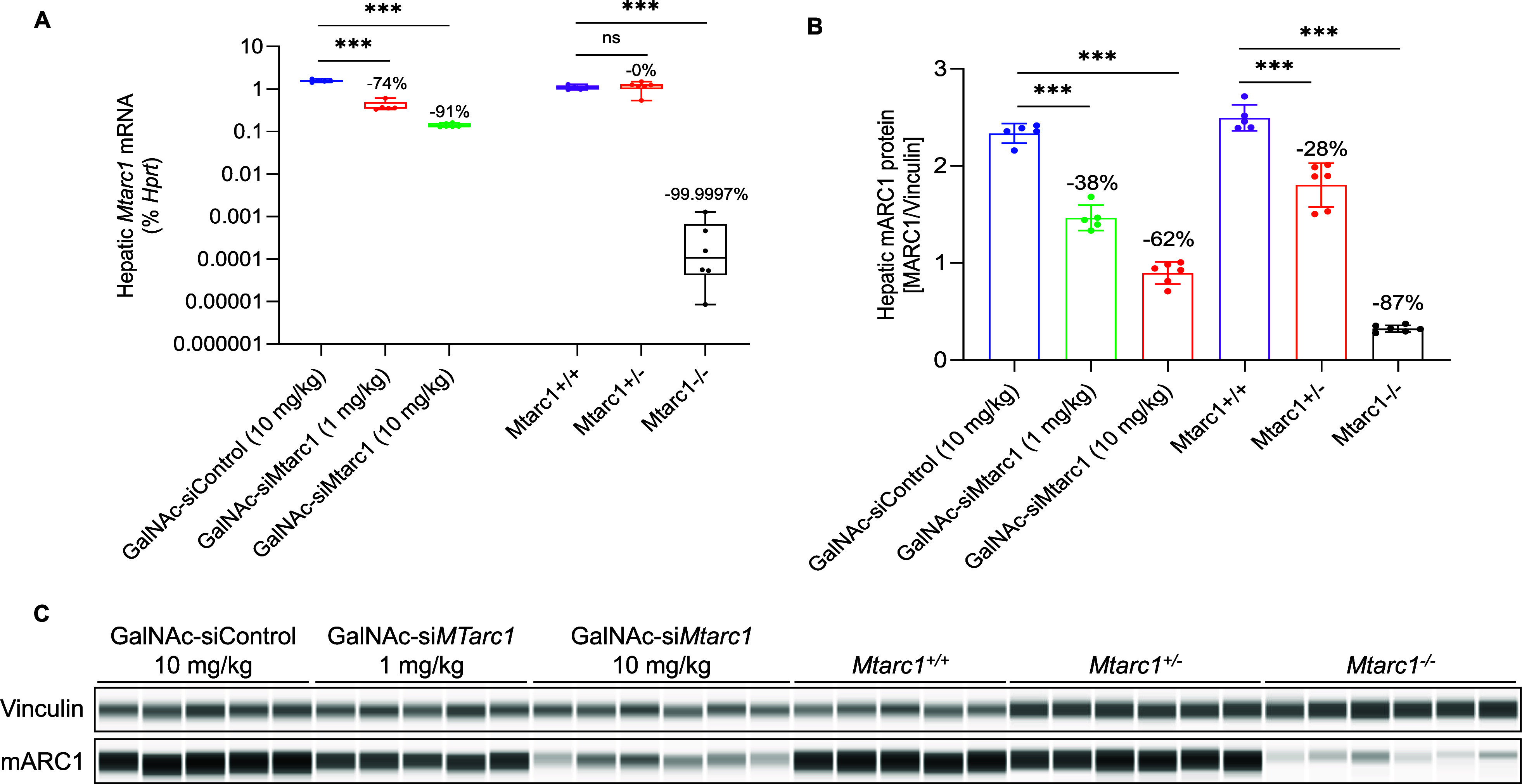
Quantification of Mtarc1 in GalNAc-siRNA-treated or genetically modified mice. (A) Hepatic Mtarc1 mRNA quantified by qPCR. (B) Ratio of hepatic mARC1 protein to vinculin quantified by immunoblotting. (C) Western blot bands for vinculin a.

To test the *in vivo* metabolism of NHU, we used both therapeutic and genetic approaches to modulate mARC1 in mice. Treatment of mice with *N*-acetylgalactosamine (GalNAc)-conjugated short interfering RNA (siRNA) (GalNAc-siMtarc1) produced a dose-dependent reduction in hepatic *Mtarc1* mRNA and protein abundance (Figure 3). Similarly, a genotype-dependent reduction in hepatic mARC1 protein abundance was observed in *Mtarc1* wild-type, knockout, or heterozygote (*Mtarc1*^+/+^, *Mtarc1*^±^, and *Mtarc1*^–/–^) mice (Figure 3). Hepatic expression of *Mtarc2* mRNA was not changed in either the GalNAc-siRNA-treated or genetically modified *Mtarc1* systems (Figure S1).

### Hydroxyurea Biotransformation

**Figure 4:**
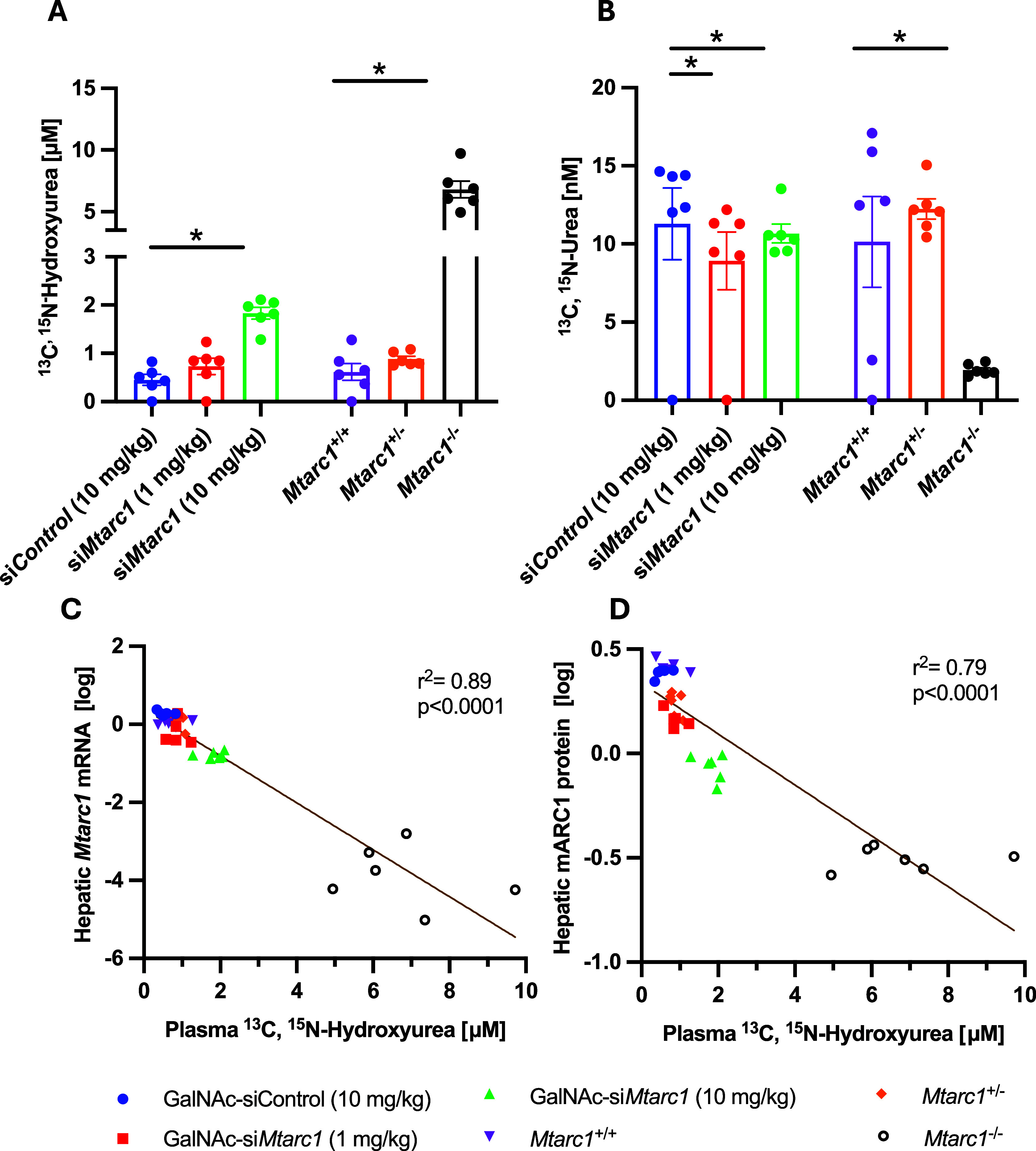
Quantification of ^13^C,^15^N-labeled NHU and urea in murine plasma 15 min after bolus application. (A)^13^C,^15^N-NHU plasma concentration. (B) ^13^C,^15^N-urea plasma concentration. (C) Correlation between hepatic Mtarc1 mRNA abundance and plasma ^13^C,^15^N-NHU concentrations. (D) Correlation between the hepatic mARC1 protein abundance and ^13^C,^15^N-NHU plasma concentrations.

To demonstrate the role of mARC1 in NHU metabolism, plasma abundance of ^13^C,^15^N-NHU was measured across *in vivo* mouse models with hepatic *Mtarc1* knockdown or knockout. Mass spectrometry analysis was used to measure the concentrations of labeled NHU and urea, permitting us to distinguish between urea derived from exogenous ^13^C,^15^N-NHU and endogenous urea production. Plasma concentrations of ^13^C,^15^N-NHU were retained with *Mtarc1* knockdown via GalNAc targeting, and the resulting product formation of ^13^C,^15^N-urea was reduced in mice with decreased mARC1 abundance (Figure 4). In *Mtarc1*^–/–^ mice, plasma abundance of ^13^C,^15^N-NHU was maintained and formation of ^13^C,^15^N-urea was reduced compared with *Mtarc1*^+/+^ mice (Figure 4). Mice with one *Mtarc1* allele (*Mtarc1*^±^) maintained similar ^13^C,^15^N-NHU levels as *Mtarc1*^+/+^ mice (Figure 4). An inverse relationship of mARC1 abundance and plasma retention of ^13^C,^15^N-hydroxyurea was observed at both the mRNA (*R*^2^ = 0.89) and protein (*R*^2^ = 0.79) level (Figure 4).

## Discussion

While the hepatic reduction of *N*-hydroxyurea to urea has been previously described as the main biotransformation pathway for this drug substance, so far, it has never been shown which specific enzyme is responsible for this reaction. The endogenous synthesis of NHU occurs in various species through arginase converting *N*^ω^-hydroxy-l-arginine to NHU and ornithine.^39^ The data presented here demonstrate that mARC1 is the main catalyst for the metabolic reduction of NHU to urea. NHU has not only been used successfully in the treatment of hematological diseases since the 1960s but is also being considered for additional indications.

*Mtarc1*-GalNac-siRNA treatment increased plasma NHU accumulation only at the high dose (10 mg/kg) and decreased plasma urea concentrations at both the low and high dose (1 and 10 mg/kg, respectively). Mtarc1 KO also had the same direction of effect on both plasma NHU and urea; homozygous KO mice had significantly increased plasma NHU and significantly decreased plasma urea, while heterozygous mice had no difference in plasma concentrations of either metabolite compared to wild-type mice. There is no evidence from this study that short-term *MTARC1* GalNAc-siRNA treatment might lead to the upregulation of alternative NHU-metabolizing pathways based on these data. Longer-term treatments might have differing effects. To elaborate on another point: liver mARC1 protein knockout was complete in the KO mice, while there was some residual liver mARC1 protein in the GalNAc-siRNA-treated mice (Figure 3B,C). There is a band remaining in the western blot of the KO mouse samples, which we attribute to the background produced by this antibody. The correlation between mARC1 protein depletion and plasma NHU clearly shows that the residual mARC1 protein in the GalNAc-siRNA-treated mice has an effect on the NHU conversion in this cohort (Figure 4D).

The key role of mARC1 in the elimination of NHU has important implications for its clinical use. For example, nonsense variants of mARC1 like Arg200Ter and Arg300Ter do occur in different populations,^32,40^ and carriers of these variants can be expected to display dramatically decreased rates of NHU elimination. Additionally, potential future therapeutic agents for prevention or treatment of liver diseases by targeting hepatic mARC1, either by siRNA approaches, proteolysis targeting chimeras (PROTAC), or traditional small-molecule inhibitors, can be expected to increase blood levels of NHU in patients who are treated with this drug. This potential for interaction must be considered early in clinical development projects.

In this study, we found that NHU is reduced by mARC1 with a very low *K*_M_ value, while mARC2 has an approximately 40 times higher *K*_M_ value and also lower turnover rates. The bolus dose administered in our *in vivo* experiments was 100 μmol × kg^–1^, which corresponds to 7.6 mg × kg^–1^, which is roughly equivalent to a single dose administered to a patient with sickle cell disease. In our present study, 15 min after injection, low micromolar NHU concentrations were found in the animal’s blood plasma. Given that the *K*_M_ value of mARC2 for NHU reduction is in the high millimolar range, we assume that mARC2 would have no relevant role in NHU metabolism *in vivo*.

The difference in NHU reduction catalyzed by mARC1 and mARC2 is somewhat unexpected, as most *N*-hydroxylated substances are reduced with similar kinetic parameters by both mARC1 and mARC2,^26^ a notable exception being *N*-oxides.^30,31^ In contrast to other molybdenum-containing enzymes, e.g., sulfite oxidase, the crystal structure of mARC1 shows the enzyme not having a well-defined substrate binding pocket.^41^ Instead, the catalytic molybdenum ion is highly solvent-exposed.^26^ This structural feature makes the highly different affinities of NHU to mARC1 and mARC2 even more surprising as it is hard to explain this difference in affinity by any specific interaction between the enzyme’s active sites and the substrate. It appears that the substrate recognition in mARC enzymes is driven solely by the chemical properties of the molybdenum active site, e.g., the redox potential, which was found to be different in mARC1 and mARC2.^42^

In mice, mARC1 is mostly expressed in the liver, whereas in humans, mARC1 is expressed more broadly in a variety of tissues.^43,44^ Therefore, the results from the animal experiments cannot be directly translated to humans. In mice, hepatocyte mARC1 is clearly the main contributor to NHU reduction, as shown by the liver-specific GalNac knockdown. The extent to which other tissues might contribute to the reduction of NHU to urea in humans cannot be estimated based on the data presented here.

Hepatocyte-specific *MTARC1* mRNA knockdown is considered by many actors as a promising strategy for treatment or prevention of liver diseases.^26,28,36^ Systemic application is always associated with a high risk of side effects and drug–drug interactions, whereas targeted therapy promises fewer undesired effects.^45^ Targeting mARC1 through a liver-specific GalNac-siRNA approach rather than a PROTAC or small-molecule inhibitor could also—to a certain extent—reduce the risk of drug–drug interactions with mARC1 substrates, e.g., NHU, as extrahepatic mARC1 would be unaffected by this approach.

As shown by the correlation of hepatic *Mtarc1* mRNA and mARC1 protein with plasma levels of NHU in the murine models (Figure 4), it is possible to indirectly detect and quantify hepatic mARC1 activity *in vivo* by monitoring NHU levels in blood plasma after an NHU bolus application. As the physiological substrate of mARC enzymes remains unknown, the concept of using exogenous substrates like NHU as marker substances for *in vivo* studies could provide a surrogate biomarker approach for target expression. For many other candidate biomarker molecules, the largely overlapping substrate profiles of mARC1 and mARC2 would complicate this approach unless both paralogues were to be targeted. While it appears that both mARC1 and mARC2 are involved in lipid metabolism in some capacity,^26^ the protein variants of mARC1 A165T, for example, have been identified as beneficial in liver disease by genome-wide association studies. Carrying this variant is associated with an improved lipid profile and a corresponding protective effect against MASLD and MASH.^26,35^ Therefore, the specific targeting of mARC1 appears to be a more desirable approach at this stage for treatment in liver diseases.

The high specificity of NHU for mARC1 makes it suitable for monitoring mARC1 activity. At the same time, we acknowledge that NHU is an active drug that has effects in patients, including many adverse effects, implying that this particular compound might not be suitable for studies with human subjects. Nonetheless, the example of NHU shows that highly specific mARC1 substrates readily reduced at detectable rates do exist. Other—yet to be discovered—specific mARC1 substrates with a high mARC1 specificity could prove to be very useful tool compounds in mARC research. The existence of highly specific mARC1 substrates further indicates that specific inhibitors of mARC1, which could serve as therapeutic drugs, are feasible in principle.

## Conclusions

The *in vitro* and *in vivo* studies presented here demonstrate that hepatic mARC1 is the main contributor to reductive NHU metabolism, while the contribution of mARC2 appears to be negligible. This finding is somewhat surprising, as previous results indicated a large overlap between the substrate spectra of both mARC paralogues.

The crucial role of mARC1 in the elimination of NHU should be taken into account with respect to known mARC1 polymorphisms and potential future drug therapies targeting mARC1.

siRNA-mediated knockdown of *MTARC1* expression in hepatocytes has been proposed as a strategy for prevention or treatment of liver diseases like MASH. Our study demonstrates that the concentration of highly selective mARC1 substrates in blood plasma can serve as a proxy for hepatic mARC1 activity, which correlates well with *MTARC1* mRNA and mARC1 protein levels in hepatocytes.

## Materials and Methods

### Purity Statement

All chemical compounds were ≥98% pure; in the case of isotopic purity was ≥99% by NMR and MS analysis.

### Ethics Statement

All animal procedures were approved by the Institutional Animal Care and Use Committee of Boehringer Ingelheim Pharmaceuticals (Ridgefield, CT, USA), which is accredited by the Association for Assessment and Accreditation of Laboratory Animal Care (AAALAC). All animal procedures were reported according to Animal Research: Reporting of *In Vivo* Experiments (ARRIVE) guidelines.^46^ All animals were housed on a 12:12 h light–dark cycle with all compound treatments and tissue collection occurring during the light cycle. Animals were socially housed with ad lib access to food and water, bedding, shelters, and nesting supplies or chewing sticks for enrichment.

### Recombinant Protein Production

Recombinant human mARC1, mARC2, CYB5B, and NB5R3 proteins were expressed in *Escherichia coli* TP1000^47^ and *E. coli* DL41^48^ and subsequently purified by affinity chromatography essentially as described previously.^49^

### *In Vitro* Enzyme Activity Assays

Kinetic parameters of the mARC-catalyzed reduction of NHU to urea were determined using a fluorescence-based activity assay.^48^ Briefly, a serial dilution series with a dilution factor of 0.67 were incubated in a 75 μL assay volume with 9.75 μg of mARC1 or mARC2, 9.75 pmol of NB5R3 (FAD), 97.5 pmol of CYB5B (heme), and 1 mM NADH cosubstrate in 20 mM MES buffer pH 6.0 at 37 °C. After a 3 min preincubation, the reaction was started with NB5R3. Enzymatic activity was measured through the decay in NADH fluorescence (λ_ex_ = 340 nm; λ_em_ = 465 nm) over 15 min.

The product of the reaction was confirmed to be urea by a secondary chemical assay (Sigma-Aldrich #MAK410) based on the reaction of urea with *o*-phthalaldehyde and *N*-(1-naphthyl)ethylenediamine (data not shown).^50^

### *Mtarc1* Genetic Mice

Mice with constitutive knockout of the *Mtarc1* gene (NCBI Transcript NM_001290273.1) were generated via CRISPR/Cas9 gene editing using the C57BL/6NTac background at Taconic Biosciences, Inc. (Rensselaer, NY). Male *Mtarc1*^+/+^, *Mtarc1*^±^, and *Mtarc1*^–/–^ mice were generated as littermates (10–12 weeks old) for these study purposes.

### GalNAc-siMtarc1

*N*-Acetylgalactosamine conjugated short interfering RNA (*GalNAc*-*siRNA*) tools were designed and synthesized at Axolabs GmbH (Kulmbach, Germany) as previously described.^35^ Male C57BL/6NTac mice (*n* = 5–6/group, 10–12 weeks old) received a single injection of 1 or 10 mg·kg^–1^ s.c. *GalNAc-siMtarc1* or *GalNAc-siControl* 7 days prior to performing the biotransformation study to achieve hepatocyte-specific knockdown.

### Hydroxyurea Biotransformation

^13^C,^15^N-Hydroxyurea (Toronto Research Chemicals, Toronto, ON; Catalog H991002, Lot 4-ESL-42-3) was prepared in saline and administered to mice as a bolus of 100 μmol/kg at 10 mL/kg via *i.v.* tail injection. Fifteen minutes later, mice were euthanized by cardiac puncture and exsanguination under anesthesia. Whole blood was processed to plasma by centrifugation in EDTA tubes, and the liver was dissected and snap-frozen into liquid nitrogen. Samples were stored at −80 °C for analyses.

### Detection by Mass Spectrometry

Plasma detection of substrate ^13^C,^15^N-hydroxyurea and product ^13^C,^15^N-urea was performed by PharmaCadence Analytical Services, LLC (Hatfield, PA). Standards of ^1^^3^C,^15^N-hydroxyurea (*N*-hydroxyurea-^13^C,^15^N_2_, CAS-Nr. 1246814-92-5) and ^1^^3^C,^15^N-Urea (urea-^13^C,^15^N_2_; CAS-Nr. 58069-83-3) were purchased from LGC Standards Ltd., USA. The internal standard urea-^13^C,^15^N_2_,^18^O (catalog CNOLM-8871-PK) was purchased from Cambridge Isotopes Laboratories, Tewksbury, MA. Plasma (5 μL) mixed with internal standard (5 μL) was precipitated with six volumes of methanol/acetonitrile at −70 °C, and the supernatant was diluted 10-fold with acetonitrile containing 0.1% formic acid. An aliquot (10 μL) was analyzed by HILIC chromatography, and analytes were measured in positive electrospray on a triple quadrupole mass spectrometer (Agilent 6495 LC-MS) in MRM acquisition mode: ^13^C,^15^N-hydroxyurea (*m*/*z* 80.0–46.3), ^13^C,^15^N-urea (*m*/*z* 64.3–46.3), and internal standard (*m*/*z* 66.3–48.3). A calibration in plasma was linear up to 3125 nM, with lower limits of quantification of 12.5 and 3.125 nM for ^13^C,^15^N-hydroxyurea and ^13^C,^15^N-urea, respectively.

### Hepatic *Mtarc1* Expression

Hepatic mRNA and protein were detected, and Mtarc1 was performed as previously described (Jones *et al.*). Primers targeting Mtarc1 (Mm01315446_m1) and Hprt (Mm03024075_m1) were obtained from Thermo Fisher Scientific (New York, NY). A polyclonal antibody targeting mARC1 was developed with a novel rabbit immunization campaign as previously published and validated.^35^ Antibodies targeting Mtarc2 (Sigma HPA017572, Lot A106167) and vinculin (ab129002, Lot GR32702836,) were obtained from Sigma-Aldrich (St. Louis, MO) and Abcam (Waltham, MA), respectively.

### Data Analysis

All data were analyzed using GraphPad Prism, Version 9 (La Jolla, CA) and Excel 2019 (Microsoft, Inc.). Group comparisons were analyzed by one-way ANOVA with Dunnett’s comparison relative to either the GalNAc-si*Control* or *MTARC1*^+/+^ group. Simple linear regressions were performed using log-transformed expression data. Data were presented as mean ± SE with individual data points represented. Significance was considered at *p* < 0.05.

## Supporting Information Available

Hepatic mRNA abundance of Mtarc2 in mice (PDF)

## Abbreviations Used

AD: Alzheimer's disease
Cyb5B: cytochrome b5B
FDA: Food and Drug Administration
mARC: mitochondrial amidoxime reducing component
NB5R3: NADH-cytochrome b5 reductase 3
NHU: *N-*hydroxyurea
NO: nitric oxide
NOAC: non-vitamin K oral vitamin K anticoagulant
MASLD: metabolic dysfunction-associated steatotic liver disease
MASH: metabolic-associated steatohepatitis
RNR: ribonucleotide reductase
SCD: sickle cell disease
PROTAC: proteolysis targeting chimera
