# Supplementary material for: mARC1 Is the Main Contributor to Metabolic Reduction of N-Hydroxyurea
Source: J Med Chem. 2024 Oct 14;67(20):18090–7. doi: 10.1021/acs.jmedchem.4c01148 (PMC11513889; doi:10.1021/acs.jmedchem.4c01148)
Supplement: Supplementary file 1 — jm4c01148_si_001.pdf [file jm4c01148_si_001.pdf]

Supporting information

mARC1 is the main contributor to metabolic reduction of *N*-hydroxyurea

Cathrin Klopp[1,2†], Xiaomei Zhang[3†], Morgan K Campbell[3], David Kvaskoff[4], Michel A Struwe[1,2], Curtis R Warren[3], Besnik Bajrami[4], Axel J Scheidig[1], Amanda K Jones[3] and Bernd Clement[2*]

1 Zoological Institute − Structural Biology, Kiel University, 24118 Kiel, Germany
2 Pharmaceutical Institute − Medicinal Chemistry, Kiel University, 24118 Kiel, Germany
3 Boehringer Ingelheim Pharmaceuticals, Inc., Ridgefield, Connecticut 06877; Department of Cardiometabolic Disease Research
4 Boehringer Ingelheim Pharma GmbH & Co., 88400 Biberach an der Riss, Germany; Department of Drug Discovery Sciences, Discovery Science Technologies (DK, BB)

*Authors to whom correspondence should be addressed:
Email: bclement@pharmazie.uni-kiel.de

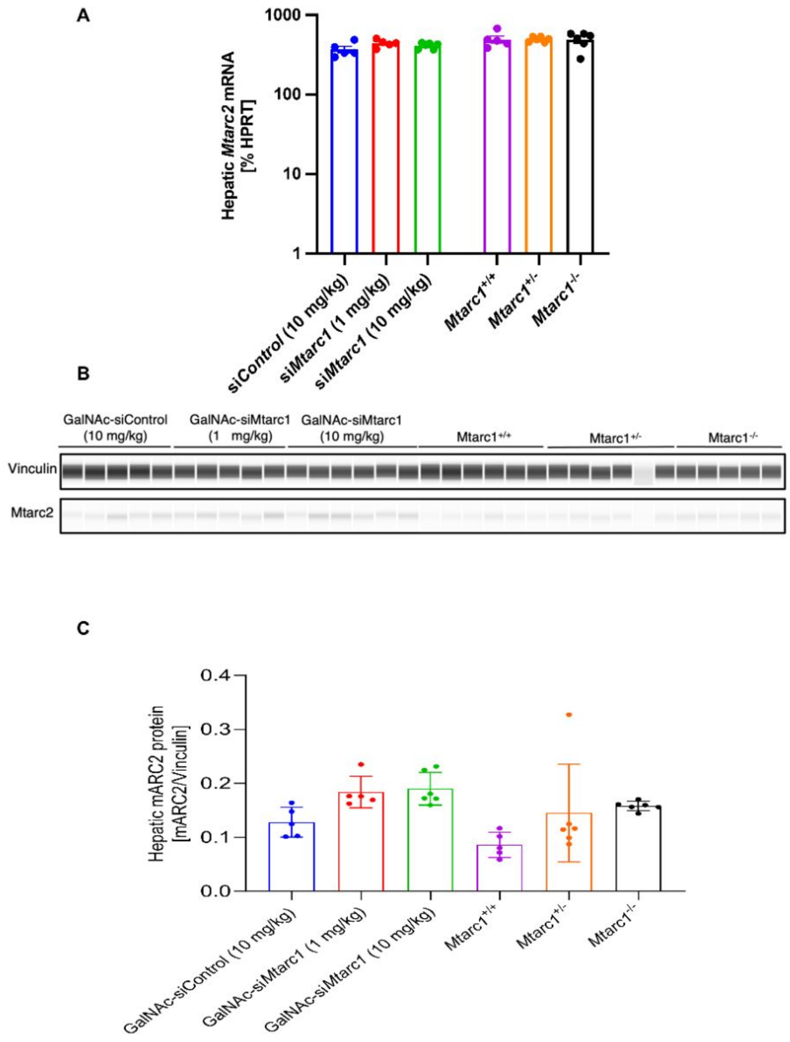

Supplementary Figure S1. Hepatic mRNA abundance of Mtarc2 in mice treated with either GalNAc-siMtarc1 or genetically modified to remove Mtarc1. Panel A: Quantification of mARC2 protein in mouse liver. Panel B: Representative immunoblot bands, Panel C: Ratio of hepatic mARC2 protein to vinculin quantified by immunoblotting.
